# CD206+ Cell Number Differentiates Influenza A (H1N1)pdm09 from Seasonal Influenza A Virus in Fatal Cases

**DOI:** 10.1155/2014/921054

**Published:** 2014-11-25

**Authors:** Heidi G. Rodriguez-Ramirez, Mario C. Salinas-Carmona, Oralia Barboza-Quintana, Americo Melo-de la Garza, Luis Angel Ceceñas-Falcon, Lilia M. Rangel-Martinez, Adrian G. Rosas-Taraco

**Affiliations:** ^1^Department of Immunology, School of Medicine and University Hospital, Universidad Autonoma de Nuevo Leon (UANL), Gonzalitos 235 Norte, Mitras Centro, 64460 Monterrey, NL, Mexico; ^2^Servicio de Anatomia Patologica y Citopatologia, School of Medicine and University Hospital, Universidad Autonoma de Nuevo Leon (UANL), Monterrey, NL, Mexico; ^3^Departamento de Anatomia Patologica, Instituto Mexicano del Seguro Social (IMSS), HGZ No. 6, San Nicolas de los Garza, NL, Mexico

## Abstract

In 2009, a new influenza A (H1N1) virus affected many persons around the world. There is an urgent need for finding biomarkers to distinguish between influenza A (H1N1)pdm09 and seasonal influenza virus. We investigated these possible biomarkers in the lung of fatal cases of confirmed influenza A (H1N1)pdm09. Cytokines (inflammatory and anti-inflammatory) and cellular markers (macrophages and lymphocytes subpopulation markers) were analyzed in lung tissue from both influenza A (H1N1)pdm09 and seasonal influenza virus. High levels of IL-17, IFN-*γ*, and TNF-*α* positive cells were identical in lung tissue from the influenza A (H1N1)pdm09 and seasonal cases when compared with healthy lung tissue (*P* < 0.05). Increased IL-4+ cells, and CD4+ and CD14+ cells were also found in high levels in both influenza A (H1N1)pdm09 and seasonal influenza virus (*P* < 0.05). Low levels of CD206+ cells (marker of alternatively activated macrophages marker in lung) were found in influenza A (H1N1)pdm09 when compared with seasonal influenza virus (*P* < 0.05), and the ratio of CD206/CD14+ cells was 2.5-fold higher in seasonal and noninfluenza group compared with influenza A (H1N1)pdm09 (*P* < 0.05). In conclusion, CD206+ cells differentiate between influenza A (H1N1)pdm09 and seasonal influenza virus in lung tissue of fatal cases.

## 1. Introduction

Influenza is a viral infectious disease recognized as a major public health problem worldwide due to high morbidity and mortality. Seasonal influenza causes more than 200,000 hospitalizations and approximately 36,000 deaths each year in the United States during the fall and winter [1]. New influenza virus outbreaks are expected every 8–41 years, similar to influenza A (H1N1)pdm09 (2009). During these outbreaks, more than 50% of the population could be infected due to the high transmissibility of the virus. Severe pneumonia is frequent in fatal cases of influenza A (H1N1)pdm09 [1] and diffuse alveolar damage (DAD) has been reported in 83% of fatal cases of influenza A (H1N1)pdm09 [2]. Immune response to influenza virus infection has been extensively studied on blood samples from patients with moderate or severe infection to explore severity markers. Previous studies showed high levels of inflammatory cytokines in sera or plasma, named “cytokine storm” or hypercytokinemia [3–5]. This exacerbated activation of the immune system may result in acute lung injury and contribute to higher rates of fatal cases. Early immune response to influenza A (H1N1)pdm09 in sera from hospitalized patients was characterized by Th1 and Th17 related cytokines, important mediator of cellular immune response and inflammation [6]. Recently, high levels of proinflammatory cytokine and chemokines were found in lung tissues from fatal cases of influenza A (H1N1)pdm09; it was one of a few reports in exploring the immunological environment in the lung [7].

However, little is known about other cell populations, as lymphocytes and macrophages in lung tissue of fatal cases, as well as cytokine expression. In the present study, we analyzed viral load, tissue damage, cytokines response, and T lymphocytes and macrophages markers in lung tissues from fatal cases of influenza A (H1N1)pdm09 and seasonal influenza. Our results presented suggest that there are no differences between influenza A (H1N1)pdm09 and seasonal influenza in viral load, cytokines response, or T lymphocytes markers but CD206 number differentiates between both influenza A (H1N1)pdm09 and seasonal influenza.

## 2. Materials and Methods

### 2.1. Patients

**Figure 1:**
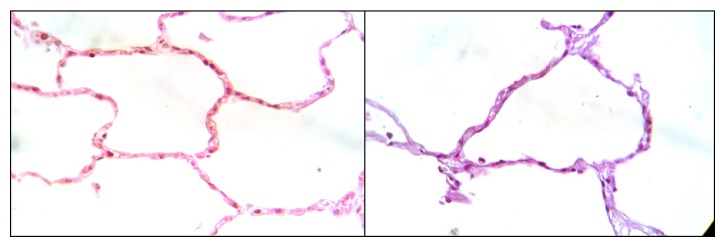
Representative examples of microscopic findings in the lung section of noninfected samples (×40).

A retrospective study was conducted on lung tissue from 72 fatal cases suspected of influenza A infection and antemortem diagnosis was pneumonia; two of them had a positive PCR for pandemic influenza. These cases were registered from August 2005 to December 2009. Five noninfected samples were included in this study; they were fatal cases of patients with carotid aneurysm, melanoma and cervix, and kidney and liver cancer, where the lung tissues of these patients show no inflammation (Figure 1). This study was approved by the Bioethics Committee of the School of Medicine at the Universidad Autonoma de Nuevo Leon (UANL) number IN10-004.

### 2.2. Virus Detection

Total RNA was isolated from formaldehyde-fixed paraffin-embedded (FFPE) lung tissues. Eight 10 *μ*m slides were placed in a microtube RNases-free, and RNA was extracted using RecoverAll Total Nucleic Acid Isolation Kit (Ambion, Austin, TX) according to the manufacturer's instructions. Finally, the concentration of RNA in each sample was determined by spectrophotometry. Influenza A [seasonal or (H1N1)pdm09] in samples was detected using the CDC's qRT-PCR protocol or PrimerDesign genesig Kit for Swine H1N1 Influenza Human Pandemic Strain (H1N1-swine) (Primer design, UK) following the manufacturer's protocol. PrimerDesign genesig Kit for Swine H1N1 Influenza Human Pandemic Strain (H1N1-swine) also was used to quantify the viral load as follows: 55°C for 10 minutes, 95°C for 8 minutes, and 50 cycles of denaturation at 95°C for 10 seconds, annealing and extension at 60°C for 60 s. Reactions were performed with 2 *μ*L of total RNA (400 ng) extract and Brilliant III Ultra-Fast QRT-PCR Master Mix (Agilent Technologies, La Jolla, CA) on the CFX96 thermocycler (Bio-Rad, Hercules, CA). Internal control (ACTB) was included in real time qRT-PCR in each sample to verify that RNA isolation and qRT-PCR were performed under the best conditions. RNA concentrations ranges were from 145.8 *μ*g/mL to 1881.2 *μ*g/mL; meanwhile, RNA purity was from 1.72 to 1.99 at 260/280 nm. Internal control amplified in all analyzed samples.

### 2.3. Histopathology Analysis

Samples were processed using standard histological protocols. Histopathological evaluation was done in tissue sections from each sample stained with hematoxylin and eosin (H&E), and lung injury score was registered as previously described [8]. Two blinded expert pathologists analyzed ten different fields of each individual sample at 40x and the average of the score was obtained from each sample.

### 2.4. *In Situ* Detection of Cytokines and Cells Markers

Paraffin embedded blocks from lung of fatal cases of influenza A patients and noninfected groups were cut in 5–7 *μ*m sections. The sections were prepared for immunohistochemistry (IHC) using standard protocols. The slides were incubated overnight at 4°C with a primary antibody against human cytokines (IL-4, IL-10, IL-17, IFN-*γ*, and TNF-*α*) or macrophages (CD14 was used for general macrophages population marker and CD206 for alternatively activated macrophages “AAM”) and lymphocytes populations (CD4 was used for T helper cells, CD8 for T cytotoxic cells, and FOXP3 for T regulatory) markers; list of antibodies is presented in Table 1. Thereafter, with another biotinylated secondary antibody, samples were incubated during 1 h at RT. The specific antibody binding reaction was amplified by incubation with ABC development system (Vector Lab, Burlingame, CA) during 30 min at RT. After the amplification step, the slides were washed and incubated again for 3 min with the chromogen substrate for HRP 3,3-diaminobenzidine (DAB) (Vector Lab, Burlingame, CA). Finally, the slides were counterstained using hematoxylin and mounted using VectaMount (Vector Lab, Burlingame, CA). The total positive cells were counted within ten different observation fields at 40x. Then, the mean of positive cells per field for each sample was calculated.

### 2.5. Statistical Analysis

The Kruskal-Wallis test was used for statistical comparisons of multiple data groups. The Mann-Whitney two-tailed unpaired *U*-test was used for statistical comparisons of two data. Calculations were performed using GraphPad Prism version 5.0 for Windows (San Diego, CA, USA). *P* values < 0.05 or less were considered significant.

## 3. Results and Discussion

### 3.1. Study Groups and Virus Detection

72 fatal cases were analyzed by qRT-PCR for seasonal and influenza A (H1N1)pdm09. Ten patients with influenza A were detected by qRT-PCR; four of them had influenza A (H1N1)pdm09. Females represented 25% of the fatal cases of influenza A (H1N1)pdm09, 83% of the seasonal influenza A, and 20% of the noninfected groups. The median age was 31.7 ± 8.75 years (range, 23–37 years) in influenza A (H1N1)pdm09 group, 37.8 ± 34.2 years (range, 24–72 years) in seasonal influenza A group, and 41.8 ± 23.3 years (range, 30–65 years) in noninfected group. Smoking was the comorbidity most frequent in fatal cases by influenza A virus followed by obesity and diabetes mellitus in the same percentages; see Table 2. Smoking, diabetes mellitus, and obesity are risk factors previously reported to influenza virus susceptibility [9, 10]. Recent study shows strong evidence of the fact that cigarette smoke extract inhibits RIG-I, IFN-*β*, and IP-10; all of them participate in innate immune response to influenza production in human lung and this may be involved in influenza susceptibility [11].

The viral loads ranged from 2 to 138 copies of RNA; M1 or N1 transcript standard curves were used for quantitative detections of influenza A (H1N1)pdm09 and seasonal influenza A virus, respectively. Recently, researchers detected influenza virus RNA from FFPE tissues obtained from autopsies using real time RT-PCR [7]. In our study, we also detected influenza virus RNA using a similar total RNA extraction kit and protocol for FFPE tissues obtained from autopsies from fatal cases suspected of influenza A infection. These findings open a new approach to study influenza virus in suspected samples, as well as the pathogenesis and infection biomarkers in human lung tissue. Studies have reported the viral load in peripheral blood, mononuclear cells, or lung aspirates from influenza virus infected patients; however, none included lung tissue [12, 13].

### 3.2. Lung Injury Score Was Not Different in Both Influenza A (H1N1)pdm09 and Seasonal Influenza A Virus

**Figure 2:**
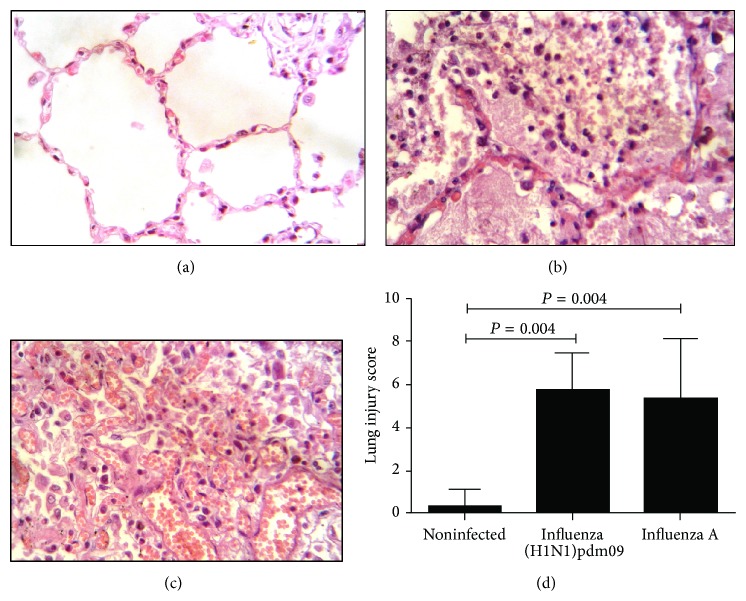
Similar lung injury scores were found in influenza groups. (a)–(c) H&E stain of three studied groups where tissue damage was evident in influenza groups. *P* < 0.05, Kruskal-Wallis test. (d) Tissue damage score showed no significant differences between influenza groups.

Lung injury is present in influenza infection [14], and similar findings were found in influenza groups as visualized in Figures 2(b)-2(c). We questioned if lung injury levels may distinguish between both influenza A (H1N1)pdm09 and seasonal influenza A virus infection. Hemorrhage, interstitial infiltrates, and perivascular aggregates were analyzed in studied groups to complete injury score. High lung injury score was found in both influenza A (H1N1)pdm09 and seasonal influenza A virus; samples were compared with noninfected lung (*P* < 0.05); however, no differences were found between influenza groups (*P* > 0.05) (Figure 2(d)).

### 3.3. Inflammatory Cytokines Were Detected in Lung Tissue

**Figure 3:**
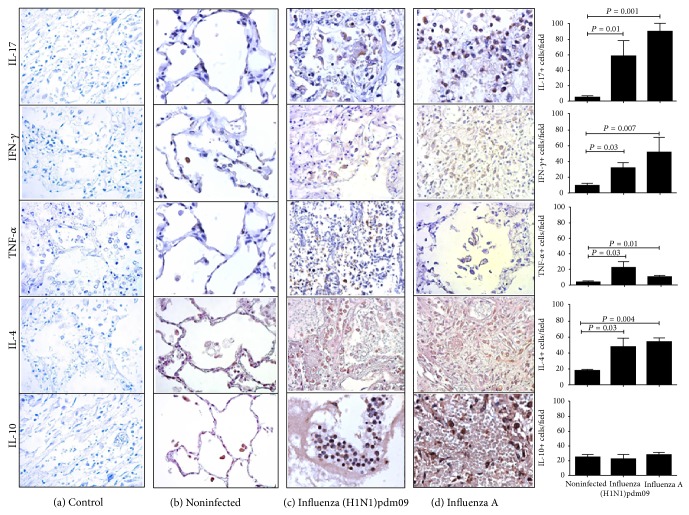
Predominant inflammatory environment cytokines were detected in influenza groups. High levels of IL-4, IL-17, IFN-*γ*, and TNF-*α* were found in lung from influenza A infected patients; meanwhile, significant differences were found in IL-10 levels (×40).

High levels of cytokine production have been previously reported in sera of severe cases of influenza A [11, 12]; we question if similar findings were occurring in lung tissue of fatal cases of influenza A and if it may be similar in influenza groups. We evaluated the inflammatory and anti-inflammatory cytokine levels. High levels of IL-17, IFN-*γ*, and TNF-*α* were found in influenza samples when compared to noninfected lung (*P* < 0.003, *P* = 0.008, and *P* = 0.009, resp., Kruskal-Wallis test). No differences were found between those cytokines levels between influenza A (H1N1)pdm09 and seasonal influenza A virus samples (*P* > 0.05) (Figure 3). The role of IL-17 and IL-17RA in acute lung injury, morbidity, and mortality induced by influenza A virus has been reported [2, 13]. In this study, IL-17A was elevated in influenza groups, and these data suggest that IL-17 released by both local and recruited cells into the lung may contribute to the acute lung injury and patient's outcomes. In Brazil, researchers found strong expression of IFN-*γ* and TNF-*α* in lung of fatal cases of influenza A (H1N1)pdm09 [15]. In agreement with our results, high levels of TNF-*α* were found in blood in three fatal cases of influenza A (H1N1)pdm09; the same phenomenon was reflected in lung tissue [15]. IL-17, IFN-*γ*, and TNF-*α* high levels are a solid evidence of the strong inflammatory response in lung from influenza infected patients; all these findings may be involved with fatal outcome.

IL-4 is a cytokine related to AAM and Th2 response [16]; in our study, we found that cytokine was elevated in both influenza A (H1N1)pdm09 and seasonal influenza A virus (*P* = 0.036, Kruskal-Wallis test) (Figure 3). Similar results were found in plasma of patients infected with influenza A (H1N1)pdm09 and seasonal influenza A [17, 18]. Differences in these results may be related to used samples (sera, plasma, and lung tissue) and sample size. Moreover, lung tissue environment reflects the real environment of influenza infection. IL-10 is an immunoregulatory cytokine and downregulates inflammatory response [19]; however, no differences were found in IL-10 level in groups included in this study (*P* > 0.05, Kruskal-Wallis test) (Figure 3). Our results suggest that the strong inflammatory response is not controlled by IL-10 in lung from influenza patients and may contribute to lung injury and fatal outcome.

### 3.4. CD4+ and CD14+ Are Recruited in Patient's Lung Tissue with Influenza A (H1N1)pdm09 and Seasonal Influenza A Virus

**Figure 4:**
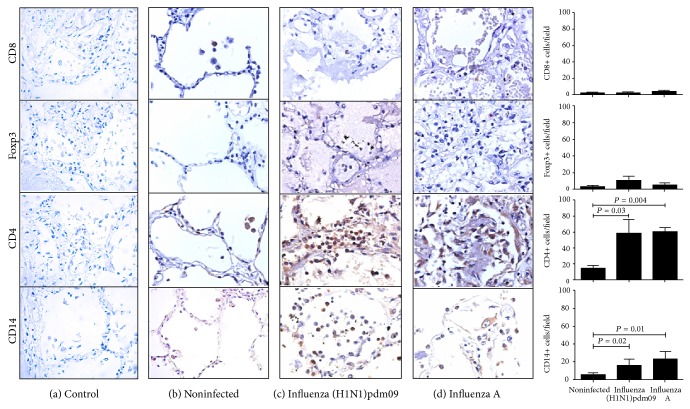
CD4+ and CD14+ cells were highly recruited in the lung of fatal cases of influenza. Representative microphotography of CD8, Foxp3, CD4, and CD14 immunohistochemistry in lung tissue of noninfected, influenza A (H1N1)pdm09, and seasonal influenza (×40). No differences were found in CD8+ cells and Foxp3+ cells. High levels of CD4+ and CD14+ cells were found in lung of influenza groups.

Although recruitment of neutrophils into the lung during influenza infection has been described, other reports have detected presence of lymphocytes population [20]. We question if there are differences in lymphocyte and macrophage subpopulations number in influenza groups, and to answer that question, we analyzed macrophages and lymphocytes markers in studied groups. CD8+IFN-*γ*+ lymphocytes have been implicated in protection against symptomatic pandemic influenza [21]. No differences were found in CD8+ cell number in fatal cases of influenza A (H1N1)pdm09, seasonal influenza A virus, and noninfected samples (*P* > 0.05, Kruskal-Wallis test) (Figure 4). These findings may explain in part why those patients succumbed to influenza infection. CD4(+) CD25(+) Foxp3+ regulatory T (Treg) lymphocyte populations are able to produce IL-10 [22]; however, in this study, IL-10 levels were not different between influenza and noninfected groups (*P* = 0.12, Kruskal-Wallis test). We evaluated Foxp3 marker in lung tissue from influenza patients and no differences were found in Foxp3 marker in the lung of influenza groups (*P* > 0.05) (Figure 4). High levels of peripheral CD4(+) CD25(+) Foxp3+ regulatory T (Treg) cells have been found in influenza A (H1N1)pdm09 [23], but in our findings, in the lung tissue, these lymphocyte populations are not increased (Figure 4). Virus specific CD4+ lymphocytes may be recruited in lung tissue [24]; here, in the present study, high levels of recruited CD4+ cells were found in influenza groups compared with noninfected tissue (*P* = 0.007, Kruskal-Wallis test), but no differences were found between fatal cases of influenza (*P* > 0.05) (Figure 4). CD4+ lymphocytes (Th1, Th2, and Th17) are producers of IFN-*γ*, IL-4, and IL-17, respectively [25]. Increased CD4+ and high levels of cytokines in lung of influenza patients support the strong inflammatory response, where Th1, Th2, and TH17 may be involved in lung injury. Patients with severe influenza A (H1N1)pdm09 infection present Th1 and Th17 hypercytokinemia in sera [6]; however, this study shows evidence that Th2 response is present in the lung together with Th1 and Th17. High levels of CD14+ macrophages were recruited in lung of fatal cases of influenza groups (*P* = 0.001, Kruskal-Wallis test); however, no differences were found between fatal cases of influenza A (H1N1)pdm09 and seasonal influenza A virus (*P* > 0.05) (Figure 4). A report suggested that CD14 has a limited role in host immune response to influenza virus infection; however, here CD14+ cells were recruited in the lung of infected patients suggesting that CD14+ cells may be producing inflammatory cytokine and in this way contributing to tissue injury as described by Pauligk et al. [26].

### 3.5. CD206+ Cells Differentiate Influenza A (H1N1)pdm09 from Seasonal Influenza A Virus

**Figure 5:**
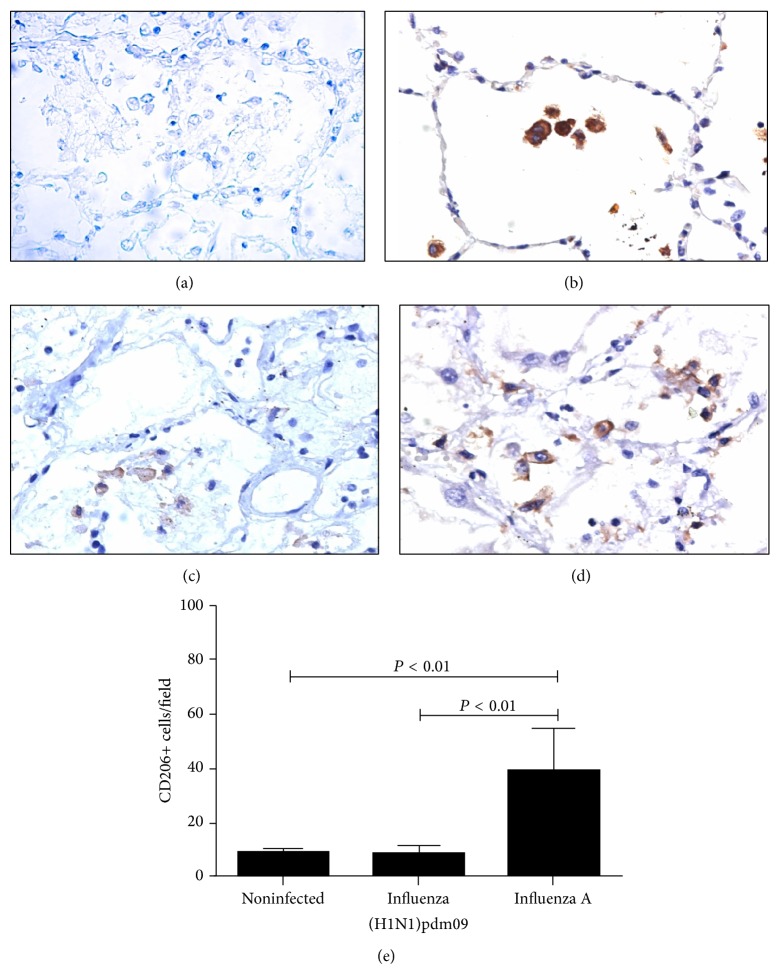
Increased CD206+ cells were found in influenza A (H1N1)pdm09. Representative microphotography of CD206 immunohistochemistry in lung tissue of (a) control, (b) noninfected, (c) influenza A (H1N1)pdm09, and (d) seasonal influenza (×40). (e) CD206+ cells were statistically significant between influenza groups.

Alveolar macrophages express predominantly an M2 phenotype [27]; CD206 is a characteristic surface marker of M2 macrophages, which has been reported in alveolar macrophages [28]. Interestingly, CD206+ cells were found in lower levels in influenza A (H1N1)pdm09 fatal cases than seasonal influenza A (*P* = 0.0007, Kruskal-Wallis test) (Figure 5). Macrophages M2 are producers of cytokines such as IL-4/IL-13 or IL-10/TGF*β*; despite the lack of difference found in IL-10 level, a report described that IL-10 and TGF*β* are necessary to improve the outcome in mice [29]. Depletion of alveolar macrophages can lead to an exacerbated inflammatory process and it is recognized that the main mechanism is the lacking of control of this process [30].

### 3.6. Imbalance of CD206 and CD14 Cells Was Found in Lung Tissue of Fatal Cases of Influenza A (H1N1)pdm09

**Figure 6:**
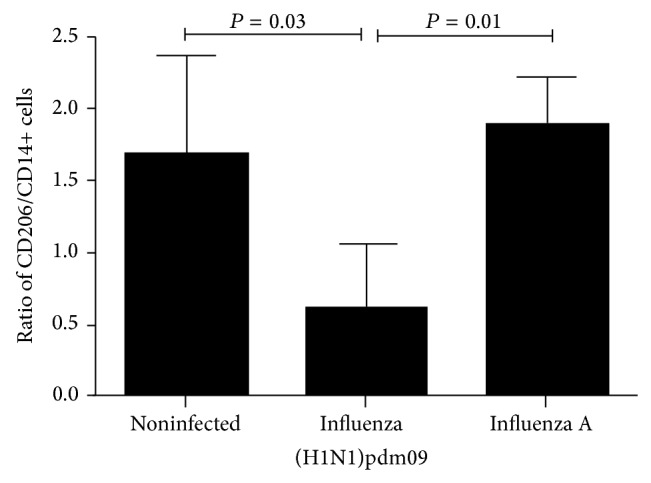
Low ratio of CD206/CD14 was found in influenza A (H1N1)pdm09 group.

In different studies, imbalance between M1 and M2 populations of macrophages has been associated with a worse prognosis in animal models [31]. Imbalance between CD206+ and CD14+ cells was evaluated assessing their ratio in lung of fatal cases of influenza. CD206/CD14+ cells ratio was 2.5-fold higher in seasonal and noninfected group than in influenza A (H1N1)pdm09 (*P* = 0.01, Kruskal-Wallis test) (Figure 6). These data suggest that there are no enough recruited healing macrophages in the lung tissue of influenza A (H1N1)pdm09 patients and this may favor inflammatory response and lung injury.

## 4. Conclusion

In this study, we demonstrated for the first time that recruited CD4+ and CD14+ cells are present in lung tissue and many may be responsible of cytokine production and in consequence lung injury in both influenza A (H1N1)pdm09 and seasonal influenza A virus in fatal cases. CD206 expression was reduced exclusively in influenza A (H1N1)pdm09; we propose that it may be used as potential biomarker to pandemic influenza and other cytokines markers may work for future therapy in severe cases of influenza A.

## Figures and Tables

**Table 1:** List of antibodies used to detect cytokines and cellular markers.

Antibodies	Dilution	Company
Rabbit polyclonal IgG anti-IL-4	0.25 *µ*g	PeproTech
Rabbit polyclonal IgG anti-IL-10	2.5 *µ*g	PeproTech
Rabbit polyclonal IgG anti-IL-17	1 : 100	Santa Cruz
Rabbit polyclonal IgG anti-IFN-*γ*	1 : 100	Santa Cruz
Rabbit polyclonal IgG anti-FOXP3	1 : 400	Santa Cruz
Rabbit polyclonal IgG anti-TNF-*α*	1 : 400	Abcam
Goat polyclonal IgG anti-CD4	5 *µ*g	R&D Systems
Mouse monoclonal IgG1 anti-CD8	1 : 50	Santa Cruz
Goat polyclonal IgG anti-CD14	2 *µ*g	Abcam
Goat polyclonal IgG anti-CD206	5 *µ*g	R&D Systems

**Table 2:** Demographic characteristics and comorbidities identified in the influenza A patients.

Patients ID	Age	Sex	Comorbidity
Seasonal influenza A patients			
4555	29	Female	Hepatitis
4586	72	Female	Chronic cardiopathy
4798	36	Male	Smoking/obesity
4807	24	Female	Smoking/diabetes mellitus
4817	29	Female	Unknown
4824	37	Female	Diabetes mellitus

Influenza A (H1N1)pdm09 patients			
4814	37	Female	Unknown
4823	23	Male	Smoking
4826	35	Male	Morbid obesity
N-13-09	31	Male	Smoking
