# Corrigendum to “CD206+ Cell Number Differentiates Influenza A (H1N1)pdm09 from Seasonal Influenza A Virus in Fatal Cases”

**DOI:** 10.1155/2020/3920701

**Published:** 2020-09-11

**Authors:** Heidi G. Rodriguez-Ramirez, Mario C. Salinas-Carmona, Oralia Barboza-Quintana, Americo Melo-de la Garza, Luis Angel Ceceñas-Falcon, Lilia M. Rangel-Martinez, Adrian G. Rosas-Taraco

**Affiliations:** ^1^Department of Immunology, School of Medicine and University Hospital, Universidad Autonoma de Nuevo Leon (UANL), Gonzalitos 235 Norte, Mitras Centro, 64460 Monterrey, NL, Mexico; ^2^Servicio de AnatomiaPatologica y Citopatologia, School of Medicine and University Hospital, Universidad Autonoma de Nuevo Leon (UANL), Monterrey, NL, Mexico; ^3^Departamento de AnatomiaPatologica, Instituto Mexicano del Seguro Social (IMSS), HGZ No. 6, San Nicolas de los Garza, NL, Mexico

In the article titled “CD206+ Cell Number Differentiates Influenza A (H1N1)pdm09 from Seasonal Influenza A Virus in Fatal Cases” [1], there was an error in Figure 3 as raised on PubPeer [2], where the first image representing IL-17was inadvertently duplicated as the first image representing TNF-*α* but displaced upwards. The corrected figure, as approved by the editorial board, is shown below.

## Figures and Tables

**Figure 1:**
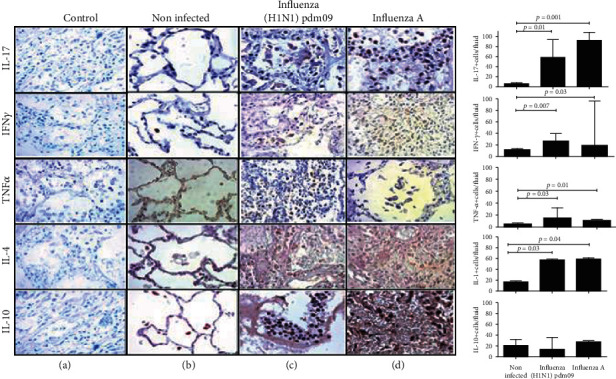
Predominant inflammatory environment cytokines were detected in influenza groups. High levels of IL-4, IL-17, IFN-*γ*, and TNF-*α* were found in lung from influenza A infected patients; meanwhile, significant differences were found in IL-10 levels (×40).
